# Supplementary material for: Droplet-based mechanical transducers modulated by the symmetry of wettability patterns
Source: Nat Commun. 2024 May 18;15:4225. doi: 10.1038/s41467-024-48538-0 (PMC11102432; doi:10.1038/s41467-024-48538-0)
Supplement: Supplementary file 1 — Supplementary Information [file 41467_2024_48538_MOESM1_ESM.pdf]

Supplementary Materials for

Droplet-based mechanical transducers modulated by the symmetry of wettability patterns

Luanluan Xue[1,2], An Li[1], Huizeng Li[1,*], Xinye Yu[1,2], Kaixuan Li[1], Renxuan Yuan[1,2], Xiao Deng[1,2], Rujun Li[1,2], Quan Liu[1,2], Yanlin Song[1,2,3,*]

1 Key Laboratory of Green Printing, CAS Research/Education Center for Excellence in Molecular Sciences, Beijing National Laboratory for Molecular Science, Institute of Chemistry, Chinese Academy of Sciences, Beijing, 100190, China

2 University of Chinese Academy of Sciences, Beijing, 100049, China

3 Xiangfu Laboratory, Jiashan, 314102, China

E-mail: lihz@iccas.ac.cn; ylsong@iccas.ac.cn

The Supplementary Materials include:

Supplementary Figures

Supplementary Discussion

Other Supplementary Materials for this manuscript include:

Videos 1 to 7 (.mov)

Description of Additional Supplementary Files

Table of Contents

1. Supplementary Figures 3

2. Supplementary Discussion 16

2.1 The design principle of the gourd pattern 16

2.2 The mechanism for continuous rotation 16

2.3 The influence of frequency on the laser trajectories 16

1. Supplementary Figures

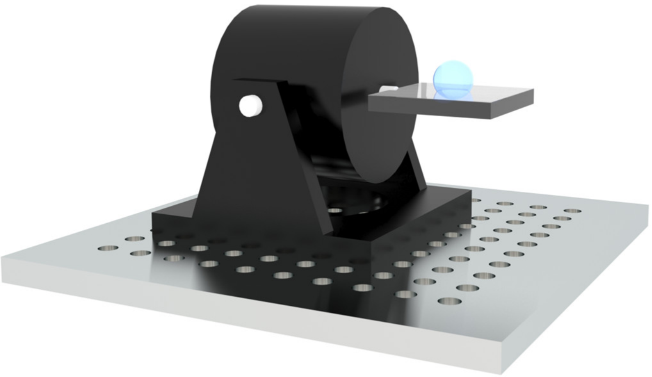

Supplementary Fig. 1 | The experimental setup. The vibration stage is fixed on an optical platform, and vibrates horizontally. The wettability patterned substrate is sticked on the vibration stage, and the steel bead/hydrogel bead/droplet is placed on it.

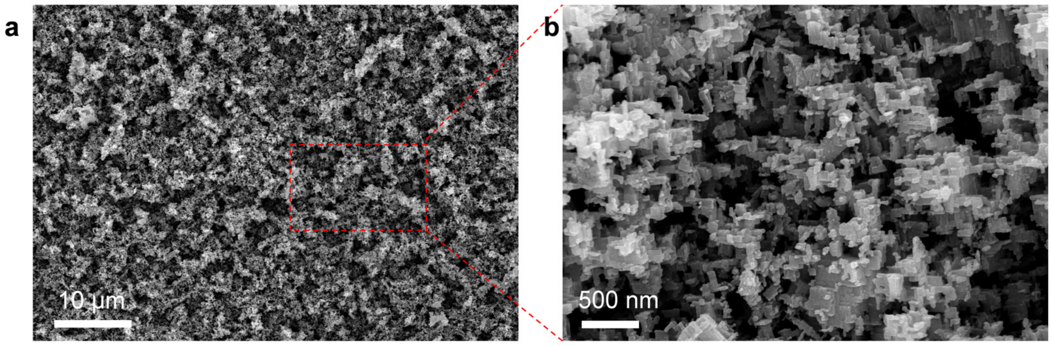

Supplementary Fig. 2 | The surface morphology of the micro-nanostructured substrate. The wettability patterned substrate is physically homogeneous.

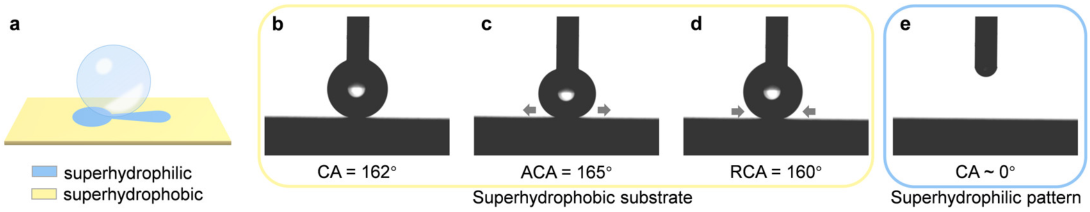

Supplementary Fig. 3 | The wettability of the chemically heterogeneous substrate. a, The scheme of the stage surface. **b-d**, The (**b**) contact angle, (**c**) advancing contact angle, and (**d**) receding contact angle of the superhydrophobic area are 162°, 165°, 160°, respectively. **e**, The contact angle of the superhydrophilic pattern ~0°.

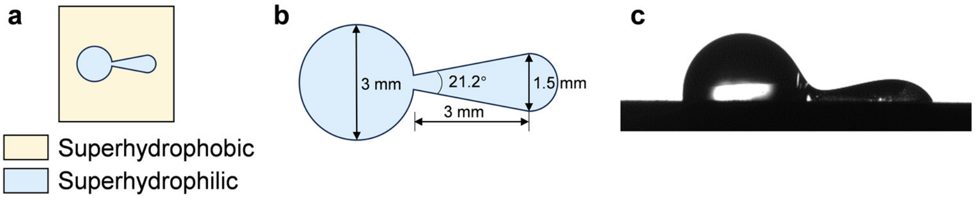

Supplementary Fig. 4 | The pattern design and the droplet morphology on the gourd pattern. **a**, The stage is superhydrophobic with a superhydrophilic gourd pattern. **b**, The pattern consists of two circles of unequal sizes (3 mm and 1.5 mm) and a trapezoid connecting channel. **c**, When a glycol droplet (V = 10 μL) rests on the wettability pattern, the distribution of the liquid reaches hydraulic equilibrium under the Laplace pressure gradient, with most of the liquid stored in the large circle. As the gourd pattern breaks the flow symmetry of the liquid, the droplet exhibits asymmetric deformation when vibrating.

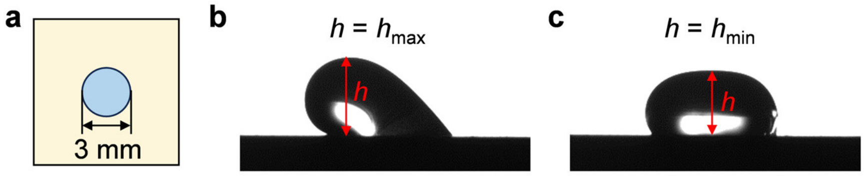

Supplementary Fig. 5 | The vibration behavior of droplet on the circle pattern. a, The pattern design of the stage. **b**, When the stage deviates to the largest displacement, the droplet is stretched to its height peak. **c**, The lowest point of the droplet occurs when the droplet is nearly symmetric and ellipsoid. With the symmetric circle pattern, the droplet exhibits symmetric deformation.

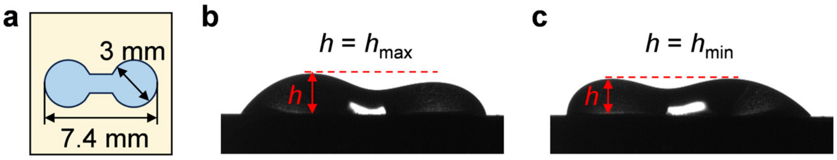

Supplementary Fig. 6 | The vibration behavior of droplet on the dumbbell pattern. a, The pattern design of the stage. **b**, When the stage deviates to the largest displacement, the liquid in the droplet accumulates in one side of the reservoir, showing the peak of its height. **c**, When the stage is at its equilibrium position, the liquid in the droplet distributes nearly equally in the two reservoirs, showing the minimum of its height. The droplet on the dumbbell pattern also shows symmetric deformation.

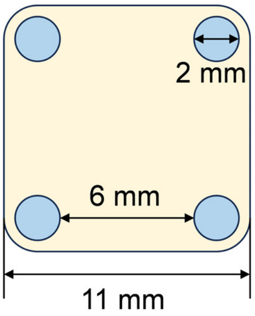

Supplementary Fig. 7 | Design of the upper plate. The upper plate is superhydrophobic with four superhydrophilic dots to fix the four droplets. The upper plate is only 15 mg in weight to minimize its influence on the droplet vibrating behavior.

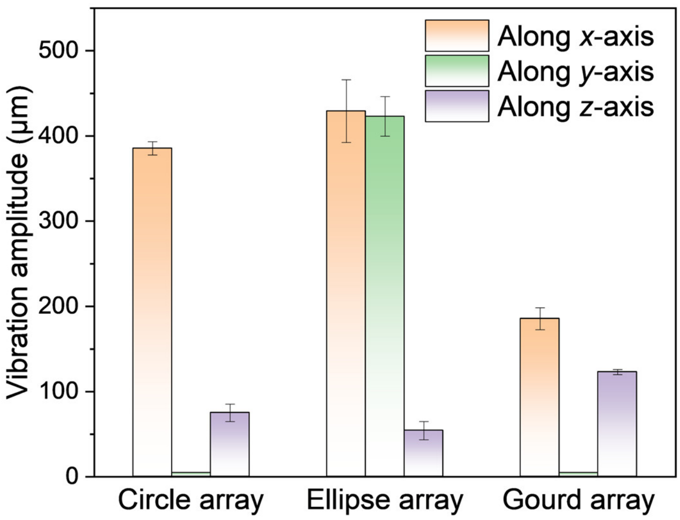

Supplementary Fig. 8 | The vibration amplitude along *x*, *y*, *z*-axis for circle array, ellipse array and gourd array. For the circle array, the translation amplitude along *x*-axis is much larger than the amplitude along *y* and *z*-axis, so we consider the translation along *x*-axis as its main motion and ignore the others. For the ellipse array, its main motion is the oblique translation along *x* and *y*-axis, while its translation along *z*-axis can be ignored. For the gourd array, the translation along *x*-axis is greatly diminished, while its translation along *y*-axis can be ignored. So, we consider its main motion as the translation along *z*-axis (The error bars represent the standard deviation, $n = 3$).

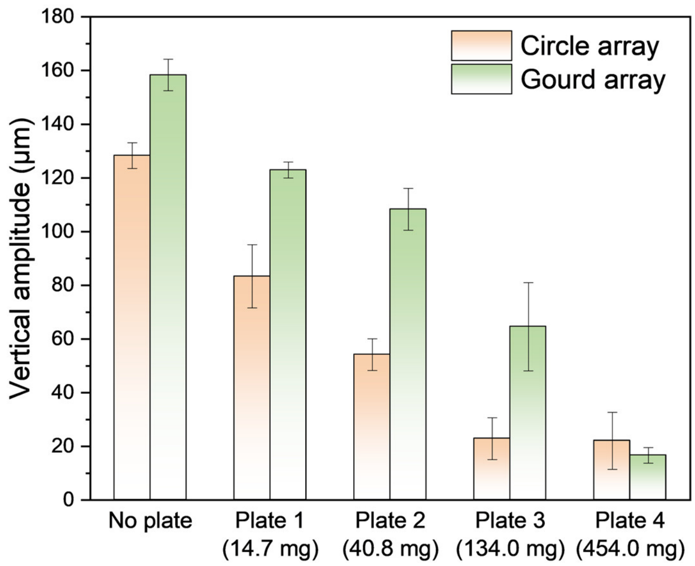

Supplementary Fig. 9 | The influence of the upper plate weight on its vertical vibration amplitude. When the weight of the upper plate increases, the vertical translation amplitude decreases rapidly. So, it is beneficial to use a heavier upper plate to suppress the vertical vibration of the circle array, while a lighter upper plate is preferred to show the vertical translation of the gourd array (The error bars represent the standard deviation, $n = 3$).

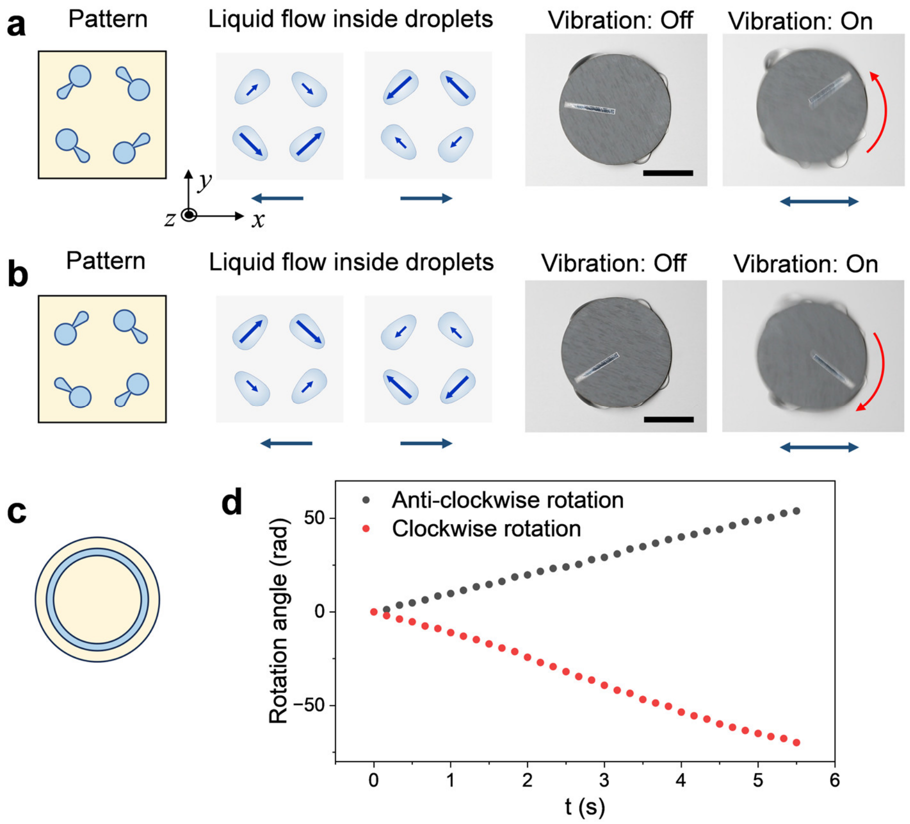

Supplementary Fig. 10 | The continuous rotation of the upper plate. a-b Anti-clockwise (**a**) and clockwise (**b**) rotation realized by gourd patterns arranged in a circle queue on the vibration stage. The length of the arrows in droplet indicates the intensity of liquid flow. **c**, The ring pattern on the upper plate. **d**, The rotation angle verse time of clockwise/anti-clockwise rotation modes. Scale bar: 5 mm.

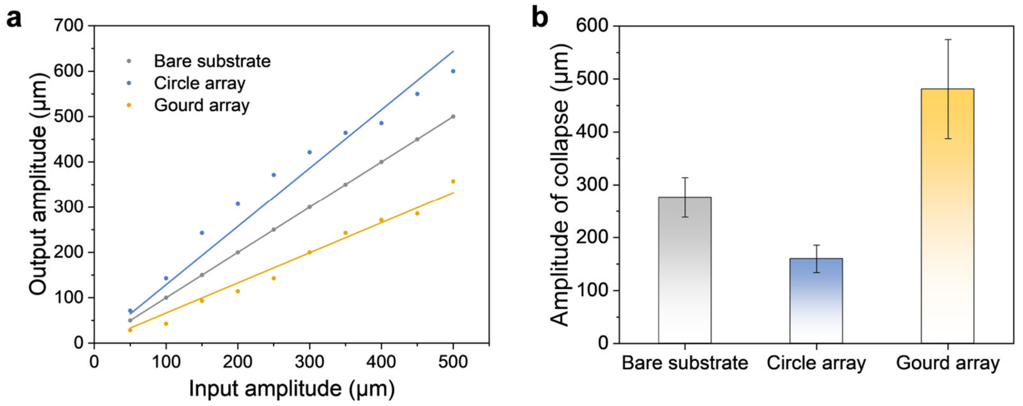

Supplementary Fig. 11 | The aseismic energy manipulation ability of different substrates. a, When the vibration frequency is 30 Hz, and the amplitude of the stage increases from 50 to 500 μm, the mechanical transducer with circle array shows enhanced vibration along *x*-axis, while the mechanical transducer with gourd array shows vibration reduction along *x*-axis. **b**, For the vibration with a frequency of 30 Hz, the threshold amplitudes of structure collapse for the bare substrate, the mechanical transducers with circle array and gourd array are 276 ± 38, 160 ± 26, 481 ± 93 μm, respectively (The error bars represent the standard deviation, $n = 5$). Compared with the bare surface, the mechanical transducer with circle array shows reduced stability, while the mechanical transducer with gourd array shows greatly enhanced aseismic capability.

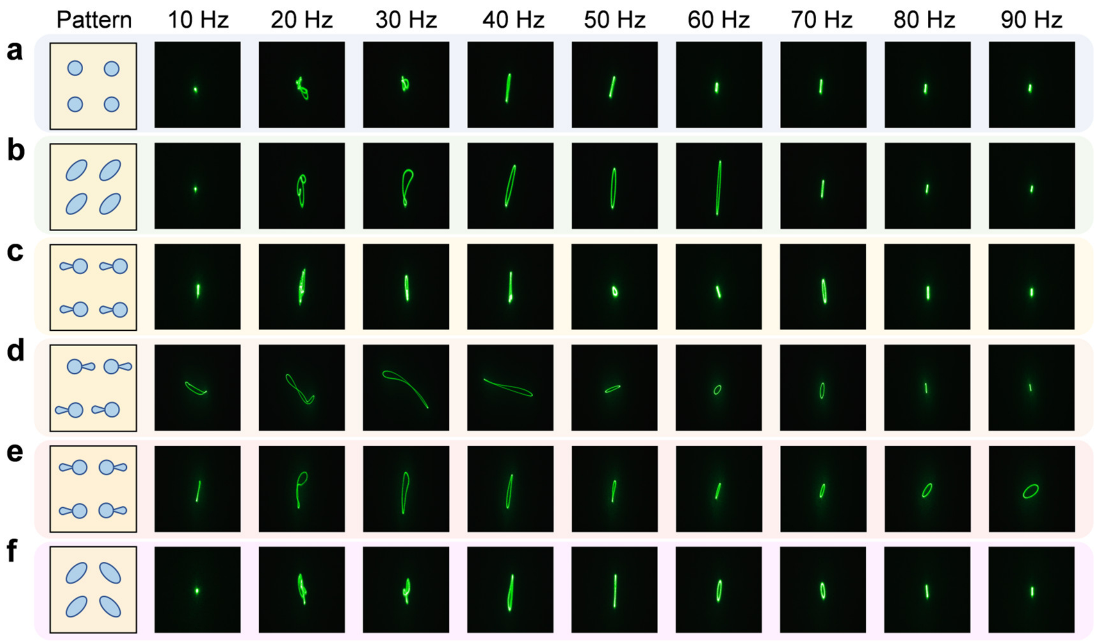

Supplementary Fig. 12 | The laser trajectory modulation achieved by different frequencies and mechanical transducers with different patterns. The frequency range is 10 Hz to 90 Hz and the patterns are (**a**) circle array, (**b**) ellipse array, (**c**) gourd array, (**d**) R-gourd array, (**e**) M-gourd array, and (**f**) M-ellipse array, respectively.

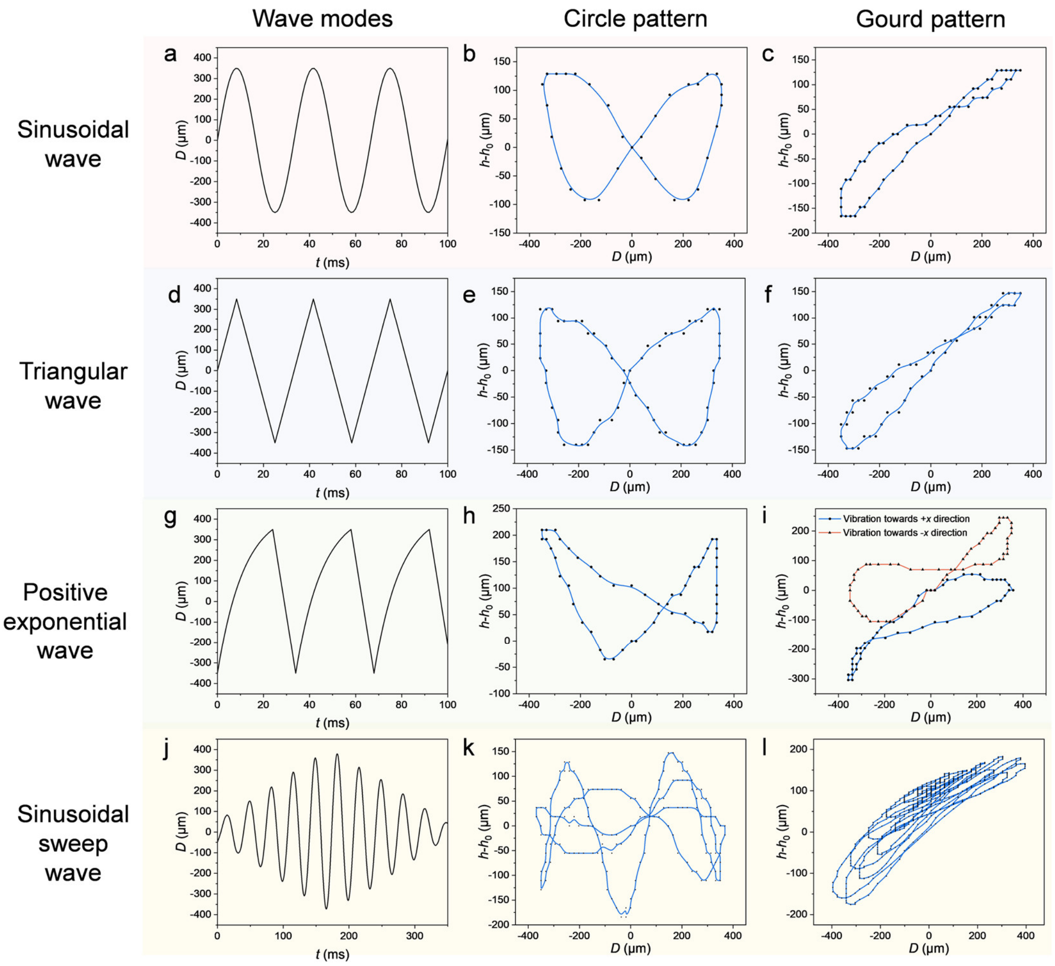

Supplementary Fig. 13 | Droplet behaviors driven by different vibration modes. a-c, The (**a**) wave mode and the corresponding droplet behavior on (**b**) circle pattern and (**c**) gourd pattern driven by a sinusoidal wave. **d-f**, The (**d**) wave mode and the corresponding droplet behavior on (**e**) circle pattern and (**f**) gourd pattern driven by a triangular wave. **g-i**, The (**g**) wave mode and the corresponding droplet behavior on (**h**) circle pattern and (**i**) gourd pattern driven by a positive exponential wave. **j-l**, The (**j**) wave mode and the corresponding droplet behavior on (**k**) circle pattern and (**l**) gourd pattern driven by a sinusoidal sweep wave.

2. Supplementary Discussion

2.1 The design principle of the gourd pattern

The design principle of the gourd pattern is to break the pattern symmetry. Thus, the flow symmetry of the liquid in droplet will be broken, and the droplet will transform the symmetric input into an asymmetric output. To have intense, asymmetric inner flow, a big circle and a small circle is designed, and a trapezoid is built in between as a flow channel. Due to the different size of the two circles, the liquid mainly lies on the large circle when static, the morphology of which can be calculated according to the Laplace pressure. In addition, the trapezoidal channel causes a Laplace pressure gradient with its direction opposite to the two circles, which facilitates the liquid flow into the small circle. As a result, when the stage vibrates, the droplet vibrates under the effect of inertia force and capillary force, shows an asymmetric flow along the pattern, indicating asymmetric output. When the stage vibrates towards the left, the liquid shunts into the small circle at the right, showing a reduced height. When the stage vibrates towards the right, and the liquid accumulates into the large circle at the left, exhibiting an expanded height.

2.2 The mechanism for continuous rotation

Besides the alternating rotation shown in Figure 3h, unidirectional rotation can also be realized by designing wettability patterns on both the stage and the upper plate. The key to continuous rotation is to achieve directional liquid flow on the stage and have continuous liquid channel on the upper plate. When the liquid flows from the big circle to the small circle, a larger Laplace pressure is created due to the large fluid volume of the big circle and the small fluid capability of the small circle. Thus, the liquid is beyond the bearing capability of the small circle and tends to flow through the liquid channel on the upper stage. On the contrary, when the liquid flows from the small circle to the big circle, a smaller Laplace pressure is created and the large circle can bear the liquid volume fluctuation. Therefore, when four gourd patterns are arranged in a circle queue (as shown in Supplementary Fig. 10a), the upper plate obtains a directional force pointing from the big circle to the small circle. Thus, a steady, continuous rotation can be generated, which has potential applications in fields including energy harvesting, micro-robotics, and micro-electro mechanical systems. The rotation direction can also be tuned by changing the orientation of the pattern (Supplementary Fig. 10b, Supplementary Video 4), with a rotational speed of 9.8 rad per second (Supplementary Fig. 10d).

2.3 The influence of frequency on the laser trajectories

The influence of frequency can be qualitatively analyzed by a simple force analysis. The droplet-plate system is subjected to three forces: the inertia force, the capillary force and the viscous force. The inertia force is proportional to $(m_{\text{plate}} + m_{\text{droplet}})D_{\max}f^2$, where m_{plate}, m_{droplet}, $D_{\max}$, and f are the mass of plate, the mass of droplet, the displacement of the stage, and the vibration frequency, respectively. The inertia force tends to keep the droplets still. The capillary force is related to the deformation of droplet and will be a function of surface energy γ, droplet radius r, and the deformation of droplet which is associated with the stage displacement $D_{\max}$ and the vibration frequency f. The capillary force tends to keep the droplets in a spherical-like shape and have the minimum surface area. The viscous force is proportional to the viscosity and the velocity gradient in the droplet, and is much smaller than the inertia force and the capillary force at a frequency below

100 Hz. Take the circle array as an example, at a low frequency (~10 Hz), the capillary force dominates to maintain the spherical shape of the droplet, so the upper plate vibrates along *x*-axis in a relatively stable way, and the laser trajectories are well consisted with the theoretical result. When the frequency increases to a range from 20 Hz to 60 Hz, the upper plate translates with a larger amplitude and is compounded with tilting, so the trajectories are vertically elongated. To be mentioned, the laser trajectory is especially sensitive to the tilting of the upper plate. Tilting of an angle less than 2° can cause the trajectory to be stretched into a line. When the frequency increases further, due to the domination of the inertia force, the vibration amplitude of the upper plate decreases, and the sizes of the laser trajectories decrease and finally shrink to a point, as shown in Supplementary Fig. 12.
