# Supplementary material for: Droplet-based mechanical transducers modulated by the symmetry of wettability patterns
Source: Nat Commun. 2024 May 18;15:4225. doi: 10.1038/s41467-024-48538-0 (PMC11102432; doi:10.1038/s41467-024-48538-0)
Supplement: Supplementary file 3 — Description of additional supplementary files [file 41467_2024_48538_MOESM3_ESM.pdf]

Description of Additional Supplementary Files

Droplet-based mechanical transducers modulated by the symmetry of wettability patterns

Luanluan Xue[1,2], An Li[1], Huizeng Li[1,*], Xinye Yu[1,2], Kaixuan Li[1], Renxuan Yuan[1,2], Xiao Deng[1,2], Rujun Li[1,2], Quan Liu[1,2], Yanlin Song[1,2,3,*]

1 Key Laboratory of Green Printing, CAS Research/Education Center for Excellence in Molecular Sciences, Beijing National Laboratory for Molecular Science, Institute of Chemistry, Chinese Academy of Sciences, Beijing, 100190, China

2 University of Chinese Academy of Sciences, Beijing, 100049, China

3 Xiangfu Laboratory, Jiashan, 314102, China

E-mail: lihz@iccas.ac.cn; ylsong@iccas.ac.cn

File Name: Supplementary Video 1

Description: The vibration transformation test of a steel bead, a hydrogel bead and a droplet. The steel bead (D = 4 mm), hydrogel bead (D = 4 mm) and droplet (V = 10 μL, glycol) are adhered/placed on the stage, and the stage vibrates with a frequency of 30 Hz and an amplitude of 350 μm. The steel bead shows no deformation, and the hydrogel bead shows symmetric deformation with its height compressed at the leftmost and rightmost position. The droplet shows alternant compression and expansion in height, indicating deformation asymmetry. The videos are recorded at 2000 fps and played in 1/40 speed of real time.

File Name: Supplementary Video 2

Description: The influence of wettability patterns on the transformation symmetry of droplets. Three types of patterns are fabricated on the stage to investigate the influence of pattern design on the transformation symmetry of droplets. The droplet on the circle pattern exhibits a symmetric bending mode, with $h_{\text{leftmost}} = h_{\text{rightmost}}$, showing symmetric output. On the dumbbell pattern, the liquid in the droplet flows symmetrically between the two circles, also showing equal h_{leftmost} and h_{leftmost}, as well as symmetric output. However, on the gourd pattern, as the symmetry of the pattern is broken, the flow symmetry is also broken. The differentiated shunt ability of the large circle and the small circle causes the alternant compression and expansion in height when the stage vibrates to the leftmost and rightmost position. Thus, $h_{\text{leftmost}} \neq h_{\text{rightmost}}$, and the droplet has an asymmetric coefficient $\eta = \frac{|h_{\text{leftmost}} - h_{\text{rightmost}}|}{h_0} = 0.23$, showing asymmetric output.

File Name: Supplementary Video 3

Description: Three-dimensional, multimodal swarm vibration behavior. By tuning the arrayed-pattern arrangement, the swarm vibration of droplets can be utilized to drive the upper plate to achieve three-dimensional, multimodal behaviors, including translation and rotation. Arrayed-pattern with translational symmetry can drive translation behavior, including translation along x-axis realized by circle array, translation along y-axis realized by ellipse array, and translation along z-axis realized by gourd array. Arrayed-pattern with translational asymmetry can drive rotation

behavior, including tilt around *x*-axis realized by R-gourd array, tilt around *y*-axis realized by M-gourd array, and rotation around *z*-axis realized by M-ellipse array.

File Name: Supplementary Video 4

Description: Transforming vibration to continuous rotation. When the gourd patterns arranged in a circle queue are fabricated on the stage, and a circular ring pattern is fabricated on the upper plate, continuous rotation can be realized. Changing the queue direction of the pattern can realize both clockwise and anti-clockwise rotation.

File Name: Supplementary Video 5

Description: Vibration management of droplet-based mechanical transducers with different patterns. Three different systems are employed, including the bare substrate, the mechanical transducer with circle pattern, and the mechanical transducer with gourd pattern. A three-layered architecture is put on top, mimicking a building. The vibration frequency is 30 Hz and the amplitude increases from 0 to 500 μm, by 50 μm each time. The architecture on the bare substrate collapses at an amplitude of 250 μm. The architecture on the mechanical transducer with circle arrayed pattern collapses at an amplitude of 150 μm, showing reduced seismic stability. By comparison, the architecture on the mechanical transducer with gourd arrayed pattern only collapses when the amplitude increases to 500 μm, showing greatly enhanced stability, which can be used in seismic energy management.

File Name: Supplementary Video 6

Description: Object transportation ability of droplet-based mechanical transducers. The mechanical transducer with M-gourd pattern causes symmetric tilting of the upper plate, so the object is localized in the center. The mechanical transducer with circle-gourd pattern causes unidirectional tilting of the upper plate, which can be used for directional transportation of the object.

File Name: Supplementary Video 7

Description: Laser modulation using the droplet-based mechanical transducers. Six patterns are used, including the circle array, ellipse array, gourd array, R-gourd array, M-gourd array, and the M-ellipse array. The vibration frequency increases from 10 Hz to 90 Hz, with a 10 Hz increase each time. When the incident laser beam is cast onto the reflector, the path of the reflected beam depends on the motion of the upper plate, and a loop trajectory composed of laser spots is formed on the background panel. We record the laser trajectory using a camera, and demonstrate that the shape and the size of the trajectory can be modulated by the pattern and the frequency.
